# Effectiveness of disease management across healthcare settings: the Multiple Sclerosis Center is a pillar for multi-disciplinary practices that promote quality of life for people with Multiple Sclerosis

**DOI:** 10.3389/fpubh.2026.1801437

**Published:** 2026-04-21

**Authors:** Eleni Grigoriadou, Eleni Polyzoidou, Christos Bakirtzis, Charis Mylonas, Evangelia Kesidou, Theano Tatsi, Elli Nteli, Natalia Konstantinidou, Charis Styliadis, Aliki Vrienniou, Georgios Papazisis, Panagiotis Bamidis, Marina Kleopatra Boziki, Nikolaos C. Grigoriadis

**Affiliations:** 1 2nd Neurological University Department, Multiple Sclerosis Center, Aristotle University of Thessaloniki, AHEPA General Hospital of Thessaloniki, Thessaloniki, Greece; 2 Laboratory of Medical Physics and Digital Innovation, Aristotle University of Thessaloniki, School of Medicine, Thessaloniki, Greece; 3 Hellenic Federation of Persons with Multiple Sclerosis (HFoPwMS), Athens, Greece; 4 Department of Clinical Pharmacology, Aristotle University of Thessaloniki, School of Medicine, Thessaloniki, Greece; 5 Hellenic Academy of Neuroimmunology (HEL.A.NI.), Thessaloniki, Greece

**Keywords:** multidisciplinary health services, Multiple Sclerosis, Multiple Sclerosis Center, personalized care, quality of life

## Abstract

**Introduction:**

Multiple Sclerosis (MS) is a chronic neurological disorder, prevalent in young adults. MS leads in disability accrual, thus affecting overall patients' quality of life. Moreover, the management of MS poses significant burden on health systems worldwide. The present study delves into the impact of different health providing settings (e.g. private office/Clinic vs. specialized MS Center) on the effectiveness of MS management, as well as on patient-reported outcomes related to the quality of life (Axis A). Moreover, the study addresses their relative effectiveness in a health crisis, such as the COVID-19 pandemic (Axis B).

**Methods:**

Data were collected based on questionnaires administered to people with MS (pwMS). Upon the pandemic and prior to the COVID-19 vaccines being available, all data were collected via online questionnaires. Since March 2021, data were collected both online and in person.

**Results:**

Overall, 776 pwMS participated in the study and answered Axis B questionnaire. Of those, 215 additionally answered Axis A questionnaire. Regarding Axis A, disease management by a specialized MS Center was associated with increased access to healthcare professionals (p < 0.001) and/or MRI examinations (*p* < 0.001) and was also linked to improved time-to-diagnosis following symptom onset, compared to the disease management in a private office/Clinic (*p* < 0.001). Regarding Axis B, specialized MS centers demonstrated remarkable adaptability during the pandemic, swiftly implementing remote care solutions to ensure continuity of care.

**Discussion:**

These findings suggest that care delivered in specialized MS centers is associated with improved access to healthcare services and better patient-reported outcomes, both under routine care conditions and during healthcare crises.

## 1 Introduction

Multiple Sclerosis (MS) is a chronic neurological demyelinating and neurodegenerative disease of the Central Nervous System (CNS) (1). MS can lead to a wide range of symptoms, including physical disability, cognitive disruption, and emotional disturbance (2). Due to its unpredictable course on a personalized level, as well as the variability in terms of the associated functional disability, MS bears a profound impact on a patient's quality of life (3). Globally, the prevalence of MS has been increasing, placing significant demands on healthcare systems that must manage the lifelong needs of those affected (4).

Managing MS effectively typically requires a multidisciplinary approach that brings together neurology, rehabilitation, and psychological care. General healthcare settings may offer basic support, but the complexity of MS often necessitates more specialized care (5). Over time, dedicated MS centers have been established to address the specific challenges posed by the disease. These specialized centers aim to provide a range of services, from advanced diagnostic tools to continuous disease monitoring, all geared toward which may contribute to improved patient outcomes. (6).

Several studies have highlighted the benefits of specialized MS centers. For example, research by Freeman et al. (7) found that patients receiving care in specialized centers had lower rates of relapse and hospitalization compared to those managed in general neurology settings. Additionally, specialized centers often engage in clinical trials and research, providing patients with access to experimental therapies that may not be available in general healthcare settings (8). This access to cutting-edge treatments and therapies can significantly improve long-term outcomes for people with MS (pwMS). Moreover, the patient-centered approach of specialized MS centers has been associated with higher patient satisfaction and better adherence to treatment. A study by Wilski et al. (9) emphasized the importance of addressing the psychological and social aspects of MS, noting that patients who receive comprehensive care that includes mental health support report better quality of life and lower levels of anxiety and depression. This holistic approach is a distinguishing feature of specialized centers and contributes to their success in managing complex, chronic conditions like MS.

The onset of the COVID-19 pandemic also brought unprecedented challenges to healthcare systems around the world, particularly in the management of chronic diseases like MS (10, 11). Routine medical care was disrupted, leading to delays in diagnosis, treatment, and follow-up care (12). For MS patients, who often require regular monitoring and timely adjustments to their treatment plans, the pandemic created significant vulnerabilities. Access to essential services like MRI scans, physical therapy, and consultations with specialists was limited due to social distancing measures and the repurposing of healthcare resources to combat the virus. We, and others, previously reported that the psychological impact of the pandemic, which heightened stress and anxiety for many, further complicated the management of chronic conditions like MS (13, 14). However, specialized MS centers were better positioned to navigate these challenges by quickly adopting telemedicine and other remote care options to ensure that patients continued to receive the necessary support (15). This adaptability highlighted the essential role of specialized centers in managing chronic diseases, particularly during times of crisis.

The present study aims to assess the effectiveness of specialized MS centers in Greece in managing MS and improving the quality of life for patients, with a special focus on the challenges posed by the COVID-19 pandemic.

## 2 Materials and methods

### 2.1 Study design

This study is an initiative of the Hellenic Academy of Neuroimmunology (HEL.A.NI.) and the 2nd Neurological University Department of the Aristotle University of Thessaloniki (AUTH) in collaboration with the Laboratory of Medical Physics and Digital Innovation. This study used a descriptive-comparative design to evaluate the effectiveness of specialized Multiple Sclerosis (MS) centers in managing disease and enhancing the quality of life for pwMS. The study was conducted in two axes as following: **Axis A:** study of the impact of different health providing settings (e.g. private office/clinic vs. specialized MS Center) on the effectiveness of MS management, as well as on patient-reported outcomes related to the quality of life and **Axis B:** study of the relative effectiveness of these health providing settings under circumstances of a health crisis, such as the COVID-19 pandemic. Both quantitative and qualitative data were collected to create a comprehensive picture of patient experiences and outcomes.

The study followed a convergent mixed-methods design, in which quantitative and qualitative data were collected during the same study period and analyzed in parallel to provide complementary insights into the research questions. Quantitative data derived from structured questionnaires were used to evaluate healthcare access, disease management characteristics, and patient-reported outcomes. Qualitative data derived from semi-structured interviews were used to explore in greater depth patients' experiences with healthcare provision settings and their perceptions of care during the COVID-19 pandemic. The qualitative findings were interpreted alongside the quantitative results to provide contextual understanding of the observed associations. This mixed-methods approach allowed the integration of patient-reported experiences with statistical analysis of healthcare access and outcomes, providing a more comprehensive understanding of the effectiveness of healthcare provision settings in the management of Multiple Sclerosis.

To address Axis A, the study compared outcomes for patients treated in specialized MS centers with those managed in general healthcare settings. Variables such as access to care, hospitalization rates, and quality of life were examined. Additionally, to address Axis B, the study explored how the pandemic influenced healthcare services and patient perceptions comparatively in specialized MS centers and in general healthcare, also by comparing outcomes upon pre - and post - (SARS-CoV-2) vaccination era. The study was in accordance to the Declaration of Helsinki and received the approval of the Bioethics' Committee of the Department of Medicine of AUTH (6.656/23-3-2021).

The study objectives were addressed through two distinct analytical axes designed to explore different research questions. Axis A examined associations between healthcare provision settings (specialized MS Centers vs. private neurological practices or other clinics) and patient-reported outcomes related to healthcare access, diagnostic procedures, and quality of life under routine healthcare conditions. Axis B focused on healthcare system responsiveness during a health crisis, specifically the COVID-19 pandemic. This axis evaluated patient-reported access to healthcare services, access to treatments, and perceived continuity of care during the pandemic period.

The two analytical axes addressed complementary but independent aspects of the study objectives and were therefore analysed separately. Axis A investigated differences in healthcare delivery structures under routine conditions, while Axis B explored healthcare access and service continuity during an external system-level disruption. No hierarchical relationship between the two analytical axes was assumed. Each analytical axis was evaluated separately using descriptive and inferential statistical analyses. This structure allowed the study to address both routine healthcare delivery conditions (Axis A) and healthcare system performance under crisis conditions (Axis B).

### 2.2 Participant recruitment

PwMS who were clinically followed by the Multiple Sclerosis Center of the 2nd University Neurology Clinic at AHEPA University General Hospital in Thessaloniki, Greece were consecutively recruited to participate in the study. An additional cohort of pwMS was invited to participate in the study by filling an online questionnaire in the site of the HELANI. These participants were invited via e-mail by the Hellenic Federation of Persons with Multiple Sclerosis (HFoPwMS). PwMS who participated in the study via the online form were asked to declare whether they were followed by a specialized MS Center, a Neurologist in a private office/other Neurological Clinic, or both. Thus, all participants were categorized based on their healthcare provision setting. Participant recruitment was conducted from April 2020 to March 2023. The anti-SARS-CoV-2 vaccines were available in Greece since February 2021, therefore, pwMS who participated before and after this date were considered as participants upon pre - and post - vaccination era, respectively. Sample size estimation was calculated using population sampling methods. Based on the estimated MS population in Greece, approximately 21,218 individuals (16), and using parameters such as a 95% confidence level and a 5% margin of error, the required sample size was determined to be 779 patients. This sample size was sufficient to draw valid conclusions about the MS population in Greece. A total of 779 people with Multiple Sclerosis participated in the study. However, not all participants completed all questionnaire components. Axis B included questions related to healthcare access and healthcare system performance during the COVID-19 pandemic and was completed by 776 participants. Axis A included additional questions related to healthcare provision settings and detailed disease management characteristics and was completed by a subset of 215 participants who provided complete responses to the relevant sections of the questionnaire. Participants with incomplete responses in specific sections were excluded only from the corresponding axis analyses. All participants provided informed consent, either in paper or online, and they were assured of their right to withdraw from the study at any time without consequence. Data confidentiality and anonymity were strictly maintained throughout the research process. In compliance with the European Union General Data Protection Regulation (GDPR), all personal data were anonymized and securely stored.

Regarding eligibility criteria and handling of missing data, participants were considered eligible for inclusion in the study if they had a confirmed diagnosis of Multiple Sclerosis and were aged 18 years or older at the time of participation. All participants were required to provide informed consent prior to participation in the study, either electronically for the online questionnaire or in written form for the in-person data collection. Participants were also required to complete the core sections of the questionnaire addressing demographic characteristics, healthcare access, and disease-related outcomes to be included in the analyses. Participants who provided incomplete responses to specific variables were not excluded from the study entirely. Instead, a case-wise exclusion approach was applied for the relevant analyses. In this approach, participants with missing data for a specific variable were excluded only from the statistical analyses involving that variable, while remaining included in other analyses for which complete data were available. This strategy allowed for the use of the maximum available dataset while minimizing the potential bias introduced by missing responses. In addition, the completeness of the collected questionnaires was reviewed prior to statistical analysis to ensure that key variables required for the primary analyses were available. Participants who did not provide sufficient information for the main study outcomes were excluded from the specific axis analyses (Axis A or Axis B) where appropriate. The proportion of missing data across variables was low and did not substantially affect the statistical analyses.

### 2.3 Data collection

Data were collected in two phases: quantitative data collection through structured questionnaires and qualitative data collection through semi-structured interviews, the latter being applicable only for participants who were followed by the MS Center of the 2nd Neurological Department of AUTH and participated in person in the study. This mixed-methods approach allowed the study to gather both measurable outcomes and deeper insights into patient experiences.

#### 2.3.1 Quantitative data collection

Structured questionnaires were administered to gather key data on healthcare access, disease progression, and quality of life. These questionnaires were completed in person during patient visits to the Neurology Clinic after obtaining informed consent. The data collected covered demographics, disease characteristics, healthcare utilization, patient satisfaction, and self-reported quality of life. Specific tools used in this phase included:

- **EuroQol 5 dimensions (EQ-5D):** A standardized instrument for assessing health-related quality of life across five dimensions: mobility, self-care, usual activities, pain/discomfort, and anxiety/depression (3).
- **Patient determined disease steps (PDDS):** A self-reported scale measuring the level of disability in MS patients, ranging from normal functioning to severe disability (17).
- **Patient-reported outcomes (PROs):** Questionnaires capturing patients' perceptions of their health outcomes, healthcare effectiveness, and overall satisfaction with care (18).

The questionnaires also included items specific to the COVID-19 pandemic, such as delays in care, access to remote care services, and the psychological impact of the pandemic on MS management.

Validated patient-reported outcome instruments were used to assess key clinical outcomes, including the EQ-5D for health-related quality of life and the Patient Determined Disease Steps (PDDS) scale for functional status. Additional questions related to healthcare access, barriers to care, and service continuity during the COVID-19 pandemic were specifically developed for the purposes of this study to capture patient experiences within the Greek healthcare system. These items were exploratory in nature and were designed to complement the validated clinical instruments used in the analysis.

#### 2.3.2 Qualitative data collection

To complement the quantitative data, semi-structured interviews were conducted with a subset of participants who agreed to share more detailed insights into their healthcare experiences. These interviews explored patient perceptions of care, challenges faced during the pandemic, and satisfaction with specialized MS centers vs. general healthcare settings. Interviews were recorded with consent and later transcribed for analysis.

### 2.4 Data analysis

The data analysis involved both quantitative and qualitative methods to provide a well-rounded understanding of the findings.

#### 2.4.1 Quantitative data analysis

SPSS (IBM SPSS Statistics 27.0) and R (The R Project for Statistical Computing 4.3.3) were used to analyze the quantitative data, identifying trends and relationships between variables. Descriptive statistics (means, medians, standard deviations) summarized the data, while inferential statistics (*t*-tests, chi-square tests) were applied where appropriate. Multiple linear regression analysis was conducted to identify variables associated with quality of life among people with Multiple Sclerosis. Functional status (PDDS score), fatigue (MFIS Physical score), and healthcare utilization variables were included as explanatory variables. Quality of life, as measured by the EQ-5D score, was used as the dependent variable. To account for potential confounding, demographic and disease-related characteristics were also included in the model, including age, disease duration, and educational level. These variables were considered important potential confounders, as previous studies have shown that demographic characteristics may influence patient-reported outcomes and access to healthcare services. Multiple linear regression analysis was conducted to examine variables associated with quality of life among people with Multiple Sclerosis. Quality of life, as measured by the EQ-5D score, was used as the dependent variable. Independent variables included functional status (PDDS score), fatigue (MFIS Physical score), healthcare provision setting (specialized MS Center vs. private practice/other neurological clinic), and healthcare utilization characteristics.

To account for potential confounding, demographic and disease-related characteristics were also included in the model, including age, disease duration, and educational level. These variables were considered important potential confounders, as demographic characteristics and disease severity may influence both healthcare access and patient-reported outcomes. The regression model therefore allowed the estimation of the association between healthcare provision setting and quality of life while adjusting for potential confounding variables.

The time period of questionnaire completion (pre - vs. post - COVID-19 vaccination era) was also considered in the descriptive comparisons in order to explore potential temporal differences in healthcare access during the pandemic.

Regression model outputs were reported as adjusted beta coefficients with corresponding 95% confidence intervals. Model diagnostics were also examined to assess the robustness of the regression results.

#### 2.4.2 Qualitative data analysis

The qualitative data from the interviews were analyzed using thematic analysis, focusing on recurring themes related to access to care, the role of specialized centers, and the challenges of managing MS during the pandemic. The findings from the qualitative analysis were integrated with the quantitative results to offer a holistic understanding of the study's outcomes.

## 3 Results

### 3.1 Participants' descriptives

A total of 779 pwMS participated in the study. Of these participants, 776 completed the questionnaire components related to Axis B analyses, while 215 participants provided complete responses to the sections relevant to Axis A analyses. Of them, 538 were women and 206 were men, with the majority having completed the questionnaire online (85%). The average age of the participants was 45.34 ± 11.32 years (Median: 39 years). Overall, pwMS followed by a specialized MS Center were younger (Median: 35 years) compared to those who were followed by a private doctor/other Neurological Clinic (Median: 45.5 years) (*p* < 0.05, Table 1). These differences in demographic characteristics between healthcare provision settings were considered in the statistical analysis in order to reduce the potential influence of confounding factors when interpreting the study findings. Regarding the time of completion of the questionnaire, ~70% of pwMS completed the questionnaire after the introduction of vaccines against COVID-19. Regarding the geographical distribution of the participants, the majority was from Greece (~98%), with the largest number of participants in the sample reportedly living in 1) the prefecture of Attica with *N* = 258 and a percentage of 33% and 2) Central Macedonia with *N* = 204 and a participation rate of 26%. Of note, the average level of education of the participants was high, as approximately 7 out of 10 (78%) reported a University degree. The most frequent disease form reported was Relapsing-Relapsing MS (*N* = 555, 71%) and the least frequent was Secondary Progressive MS (*N* = 64, 8%). It is noted that 8% responded that they did not know/did not answer the question regarding their disease form. The majority (~81%) of the participants were receiving MS-specific disease-modifying treatment at the time of their participation in the study. The most frequently used disease-modifying treatments in the participant population were dimethyl fumarate (*N* = 122, 20%), interferon-β (*N* = 114, 19%) and fingolimod (*N* = 97, 16%) (data not shown).

**Table 1:** Age distribution across healthcare provision settings.

Healthcare provision setting	Median age (IQR)	*p*-value
MS center	35.0 (30.0–45.5)	< 0.05
Private office/other neurological clinic	45.5 (38.0–51.0)	
Both	43.0 (37.0–52.0)	
Total	39.0 (35.0–50.0)	

MS, Multiple Sclerosis.

**Figure 1:**
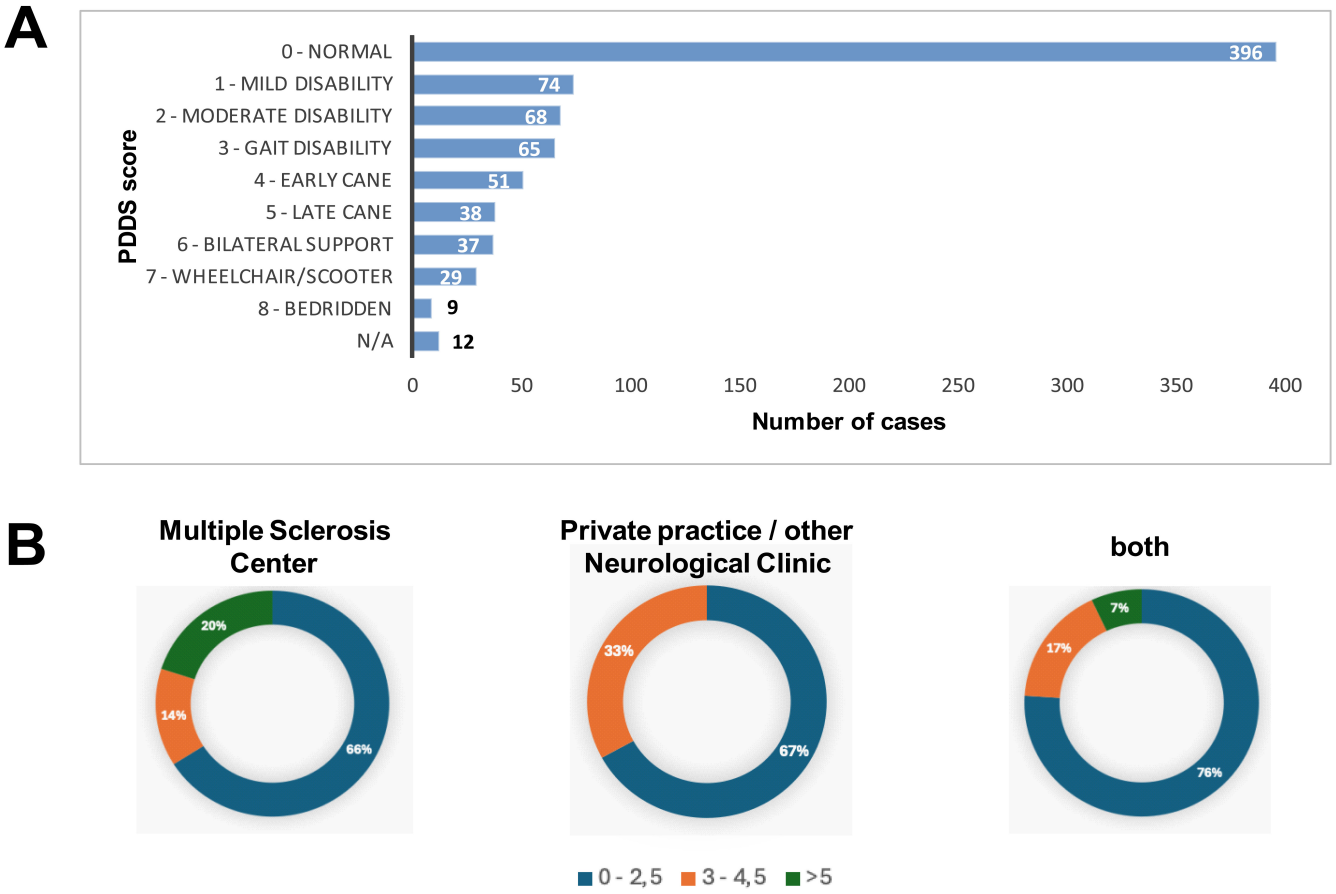
Distribution of participants based on degree of disability (self-reported outcome). Distribution of participants based on degree of disability (self-reported outcome) in total **(A)** and across different healthcare provision settings **(B)**. **(A)** Bars represent number of cases. X axis represents range in number of cases. Y axis represents PDDS total scores. **(B)** color annotations represent PDDS score range (blue: 0-2,5; orange: 3-4,5; green: >5) Percentages represent the relative frequency of cases per PDDS score range accross healthcare provision settings. PDDS: Patient-Determined Disease Steps rating scale. N/A, non-available.

For the pwMS who were followed by the MS Center of the 2nd Neurological Department of AUTH and for whom EDSS was available, a significant percentage of participants (48%) had EDSS scores between 1.5 and 2, while 29% had scores >3 and 13% had scores >6. The mean score on the scale was 2.70. In Figure 1, data regarding the self-reported degree of disability (PDDS) for all pwMS are presented. More specifically: 51% of the participants reported normal status, 9% of the participants reported mild disability, and 8% of the participants reported moderate disability, without limitation in walking ability. It is noted that a higher percentage of pwMS with increased self-reported disability (PDSS > 5) (20%) were followed in a MS Center, that is, the pwMS who were followed by the MS Center of the 2nd Neurological Department of AUTH, compared to those who were followed in a private practice/other Neurological clinic (0) and to those who stated that they were followed by both healthcare provision settings (7%), as answered in the online form. This observation may indicate an increased frequency of preference for follow-up by an MS Center in stages of the disease with increased needs for the use of health services due to the accumulation of disability and the chronicity of symptoms. However, caution is required in interpreting these results, as a percentage of patients with an increased degree of disability in the context of MS may present a reduced use of digital tools (due to difficulty using upper limbs, cognitive impairment, etc.), which makes it difficult to participate in the study via online completion of the questionnaire.

### 3.2 Axis A: effectiveness of disease management across different healthcare provision settings.

#### 3.2.1 Access to healthcare professionals/facilities/procedures

As shown in Table 2, there was a tendency for significant difference (*p* = 0.05) in patients' access to a doctor or health professional in the last 12 months from the completion of the questionnaire for MS-related issues, other than a neurologist, depending on the monitoring structure (in a MS Center, in a private practice/other Neurological Clinic or in both). More specifically, a percentage of 70% faced obstacles in accessing the doctor or health professional they needed for MS-related issues when they were monitored by a private practice/other Neurological Clinic, compared to 25% when they were monitored by an MS Center. Barriers to access were encountered by the majority of patients not attending exclusively an MS Center (*N* = 76, 77%), while the majority of patients (*N* = 69 or 75%) attending MS Center did not encounter barriers to accessing a health professional for MS-related issues. Additionally, patients who utilized both private and public MS Center services experienced better outcomes, with 53% reporting no barriers. The difference was more prominent when only pwMS followed either by an MS Center or a private practice/other Neurological Clinic were directly compared. A notable proportion — 75% of patients in specialized MS centers—reported no barriers in accessing healthcare professionals. This is a stark contrast to the 30% of patients in private practice/other Neurological Clinic who reported the same level of access. These differences were statistically significant (*p* < 0.001), confirming that specialized MS centers offer more consistent access to critical healthcare services. The number of healthcare professionals consulted was used as an indicator of healthcare utilization and access to different types of services rather than as a direct measure of coordinated multidisciplinary care.

**Table 2:** Obstacles/barriers in (a) visiting a doctor or healthcare professional for issues related to multiple sclerosis, (b) undergoing MRI examination as requested by the treating neurologist in the last 12 months and (c) need for hospitalization upon disease onset, across different healthcare provision settings and across two different types of comparison.

Answer	MS center *N* (%)	Private doctor/clinic *N* (%)	Both *N* (%)	Total *N* (%)	*p*-value
(a) Q: In the past 12 months, have you faced any obstacles/barrier in visiting a doctor or healthcare professional for issues related to multiple sclerosis, apart from your neurologist?					
No	69 (75%)	19 (30%)	28 (47%)	116 (54%)	0.05^*^; < 0.001^**^
Yes	23 (25%)	45 (70%)	31 (53%)	99 (46%)	
Total	92	64	59	215	
(b) Q: In the past 12 months, did you have an MRI as many times as requested by your neurologist or the neurologists currently treating you?					
No	5 (5%)	13 (20%)	12 (20%)	30 (14%)	**0.073** ^*^ **; 0.05** ^**^
Yes	87 (95%)	51 (80%)	47 (80%)	185 (86%)	
Total	92	64	59	215	
(c) Q: Did you need to be hospitalized due to your first symptom?					
No	75 (82%)	34 (53%)	38 (64%)	137 (64%)	< 0.05^*^; < 0.05^**^
Yes	17 (18%)	30 (47%)	21 (36%)	78 (36%)	
Total	92	64	59	215	

Q, question; MS, Multiple Sclerosis; pwMS, people with Multiple Sclerosis; N/A, non-applicable.

^*^Overall comparison;

^**^Comparison between pwMS followed either by an MS Center or a private practice / other Neurological Clinic. Percentages denote proportion over total N per healthcare provision setting.

Of all participants, the majority (*N* = 185/215, 86%) reported full access to MRI in the last 12 months. However, it is noted that a greater percentage (95%) of patients who were followed by a MS Center, compared to those who were followed by a private practice/ other Neurological Clinic (80%) and compared to those who were followed by both (80%), reported full access to MRI in the last 12 months (Table 2). The difference was more prominent when only pwMS followed either by an MS Center or a private practice/other Neurological Clinic were directly compared, with only 5% of patients visiting an MS Center experiencing obstacles. In contrast, 1 in 5 patients (20%) visiting a private practice/other Neurological Clinic faced obstacles in performing the required MRI scan(s). The data indicate that patients followed in specialized MS centers reported fewer barriers to timely diagnostic and/or follow-up procedures, which is crucial for managing disease evolution and for adjusting treatment plans accordingly.

The need for hospitalization upon disease onset was also addressed comparatively across healthcare provision settings. Only 18% of patients treated at specialized MS centers required hospitalization following the onset of their symptoms, compared to 47% of those treated in private practice/other Neurological Clinic and 36% of those reportedly followed by both (*p* < 0.05). However, this observation should be interpreted with caution. Patients followed in specialized MS centers are often referred after the initial diagnostic evaluation has been performed in other healthcare settings. Therefore, previous hospitalizations related to diagnostic procedures or initial disease evaluation may have occurred before referral to the specialized center and may not be fully captured in the current analysis. This difference was also evident when comparing only patients who were followed at a MS Center, vs. patients who were followed at a private practice/other Neurological Clinic (*p* < 0.05) (Table 2). We hereby emphasize on the heterogeneity of the population monitored by a specialized MS Center with regard to diagnostic investigation, as patients are often referred by Neurologists in private practices or other healthcare provision settings, at a later time and after the diagnosis of MS. It is often observed that patients referred to the MS Center have initially received a diagnosis of MS based on clinical and radiological criteria, without the conduction of a lumbar puncture (LP). LP is a procedure that requires hospitalization, being, however, not mandatory for the diagnosis of MS. In the frame of the present study, the full disease history regarding the accommodation of pwMS to the MS Center by referring Neurologists was not addressed, therefore, the analysis does not allow further investigation of this parameter.

#### 3.2.2 Diagnosis and disease management

The study also examined the impact of the healthcare provision setting on the management of the disease, particularly in terms of regular monitoring, follow-up care, and access to treatment options.

In the setting of regular follow-up, pwMS followed by a specialized MS Center reportedly underwent more frequent MRI monitoring. As shown in Table 3, a statistically significant difference (*p* < 0.05) was found in the number of MRI scans performed by patients within the last year depending on the MS follow-up structure. It is noted that approximately half of the patients followed in a specialized MS Center underwent at least 2 MRI scans within the last year of follow-up (47%, *N* = 43/92), compared to patients followed in a private doctor/other Neurological Clinic where the majority of patients underwent 1 or no MRI scans within the last year of follow-up (75%, *N* = 48/64). This observation can be interpreted as follows: pwMS are often referred to the MS Center in need of an alteration in the disease-modifying therapy and change of their treatment plan, either due to non-response to previous immunomodulatory therapies (breakthrough disease) and the need for further treatment escalation, or due to adverse reactions to previous immunomodulatory therapies. In both cases, these are patients in whom the monitoring protocol requires more frequent MRI imaging within the year, more than once. A lower average frequency of MRI (less than once/year, which is the recommended annual check-up frequency) for patients who were monitored by a private practice/other Neurological Clinic, is also noted.

**Table 3:** Frequency of MRI examination in the past 12 months across different healthcare provision settings and across two different types of comparison.

Q: How many times did you have an MRI in the past 12 months?					
Nr of MRI examinations	MS center *N* (%)	Private doctor/clinic *N* (%)	Both *N* (%)	Total *N* (%)	*p*-value
0	4 (4%)	8 (13%)	5 (8%)	17 (8%)	< 0.05^*^; < 0.001^**^
1	45 (49%)	40 (63%)	29 (49%)	114 (53%)	
2	31 (34%)	12 (19%)	18 (31%)	61 (28%)	
>2	12 (13%)	4 (6%)	7 (12%)	23 (11%)	
Total	92	64	59	215	

Q, question; MRI, Magnetic Resonance Imaging; MS, Multiple Sclerosis; pwMS, people with Multiple Sclerosis; N/A, non-applicable.

^*^Overall comparison;

^**^Comparison between pwMS followed either by an MS Center or a private practice / other Neurological Clinic. Percentages denote proportion over total N per healthcare provision setting.

Considering the multidisciplinary approach advocated in the management of MS, the present study also addressed the average number of healthcare professionals apart from the treating Neurologist that pwMS visited in the last 12 months, across different healthcare provision settings. A significant finding is that 30% of patients monitored at a specialized MS Center visited more than two doctors for issues related to their condition, while more than 50% of those monitored by a private doctor/other Neurological Clinic (*N* = 34 or 53%) did not visit other healthcare professionals apart from the treating Neurologist for MS-related issues (*p* < 0.05, Table 4). This observation may reflect differences in healthcare organization and access to multidisciplinary services among patients followed in MS centers and the management of MS-related chronic symptoms, through the engagement and the collaboration with a network of health professionals of several specialties, in addition to the Neurologist. Of note, the present study did not address whether the healthcare professionals, other than the Neurologist, were contacted in the frame of an MS-Center-coordinated network, or by the patients independently. Although the findings should be interpreted with caution, a possible explanation may be increased rate of referral to other specialists by the treating Neurologist, based on increased alertness towards holistic disease management. More outcomes designed to address referral patterns and communication pathways among healthcare professionals are needed in order to further investigate this assumption.

**Table 4:** Number of healthcare professionals apart from the treating Neurologist visited in the last 12 months, across different healthcare provision settings.

Q: Apart from the neurologist or neurologists currently treating you, which and how many of the following healthcare professionals did you visit in the past 12 months for the management of Multiple Sclerosis and its effects?					
Nr of healthcare professionals	MS center *N* (%)	Private doctor/clinic *N* (%)	Both *N* (%)	Total *N* (%)	*p*-value
0	35 (38%)	34 (53%)	24 (41%)	93 (43%)	< 0.05
1	29 (32%)	16 (25%)	19 (32%)	64 (30%)	
2	13 (14%)	9 (14%)	9 (15%)	31 (14%)	
≥3	15 (16%)	5 (8%)	7 (12%)	27 (13%)	
Total	92	64	59	215	

MS, Multiple Sclerosis. Numbers denote how many healthcare professionals were visited, apart from the treating Neurologist for the management of Multiple Sclerosis. Percentages denote proportion over total N per healthcare provision setting. Data regarding type of healthcare professionals are not shown.

It is noted that the time from onset of MS to diagnosis was reportedly < 30 days in 65% of patients followed by a MS Center vs. 25% of patients followed by a private practice/other Neurological Clinic. The distribution was statistically significant in the overall comparison described in Table 5 (*p* = < 0.001), as well as when the comparison concerned only patients followed by a MS Center vs. patients followed by a private practice/other Neurological Clinic (*p* = < 0.001).

**Table 5:** Time between the initial seeking of medical care and the diagnosis of Multiple Sclerosis across different healthcare provision settings.

Q: From the moment you sought medical care, how much time passed until the diagnosis of Multiple Sclerosis?					
Healthcare setting	<30 Days *N* (%)	<12 Months *N* (%)	Years (≥1) *N* (%)	Total *N* (%)	*p*
In a private practice/clinic	16 (25%)	30 (47%)	18 (28%)	64	< 0.001
In an MS center	59 (65%)	26 (28%)	7 (9%)	92	
Both	15 (25%)	35 (56%)	11 (19%)	59	
Σ*νoλo*	90	89	36	215	

MS, Multiple Sclerosis. Percentages denote proportion over total N per healthcare provision setting.

#### 3.2.3 Quality of life - patient-reported outcomes

The EQ-5D tool revealed that patients in specialized centers faced fewer limitations in mobility, self-care, and daily activities, along with lower levels of pain, discomfort, and anxiety. These differences were statistically significant (*p* < 0.05), suggesting that care in specialized MS centers is associated with better patient-reported quality-of-life outcomes. The present study also addressed how changes in the functional status of MS patients would impact their quality of life. Regression coefficients were converted into percentage changes to facilitate interpretation of the magnitude of associations between predictors and patient-reported quality of life outcomes. This analysis was conducted separately for patients monitored within a specialized MS Center and for those followed by a private doctor or other Neurological Clinic. Specifically: for patients monitored within a specialized MS Center: if the EQ-5D score increases by one unit, the quality of life of patients increases by 8.7%. Conversely, if the MFIS Physical score decreases by 1 unit, the quality of life increases by 7.1%. For patients monitored by a private doctor or Clinic: if the EQ-5D score increases by one unit, the quality of life improves by 4.2%. If the MFIS Physical score decreases by 1 unit, the quality of life improves by 3.8% (Table 6). The reported percentages reflect the relative change in predicted quality-of-life scores derived from the regression coefficients of the model. Other variables used in the model influenced the quality of life in MS patients only numerically without statistical significance. Tools including the EQ-5D, MFIS Physical, and PDSS can be considered important predictive factors for the quality of life in pwMS. Quality of life reduction was strongly correlated with patient-reported outcomes (EQ-5D and MFIS Physical), whereas correlations with clinical parameters were weak (Tables 7, 8).

**Table 6:** Correlation between patient QoL and EQ-5D and MFIS Physical parameters for patients monitored within a specialized MS Center and in a private office / other neurological clinic.

Correlation between patient QoL and EQ-5D and MFIS Physical parameters for patients monitored within a specialized MS Center.						
QoL	EQ-5D					
	0–5.0	5.1–8.0	8.1–11.0	11.1–14.0	14.1–18.0	*P*-value
	18.7%	42.9%	64.4%	86.6%	100%	< 0.001
	MFIS physical					
	0–1	2–5	6–8	9–10	–	*P*-value
	7.9%	36,8%	76.7%	100%	–	< 0.001
Correlation between patient QoL and EQ-5D and MFIS Physical parameters for patients monitored in a private office/other neurological clinic						
QoL	EQ-5D					
	0–5.0	5.1–8.0	8.1–11.0	11.1–14.0	14.1–18.0	*P*-value
	12.4%	34.1%	56.3%	78.2%	100%	< 0.001
	MFIS physical					
	0–9.0	9.1–18.0	18.1–27.0	27.1–36.0	–	*P*-value
	5.1%	27,7%	62.3%	100%	–	< 0.001

QoL, quality of life; EQ-5D, EuroQol Questionnaire; MFIS, Modified Fatigue Impact Scale; MS, Multiple Sclerosis.

**Table 7:** Multivariate linear regression analysis of factors associated with quality of life (EQ-5D).

Predictor	β coefficient	95% CI	*p*-value
Age (years)	−0.010	−0.021–0.001	0.072
Disease duration (years)	−0.002	−0.009–0.005	0.612
Educational level (university vs non-university)	0.061	0.014–0.108	0.012
EDSS score	−0.118	−0.166-−0.070	< 0.001
MFIS physical score	−0.094	−0.121-−0.067	< 0.001
Healthcare setting (MS Center vs private clinic)	0.069	0.018–0.120	0.008

Model statistics: N = 776; R^2^ = 0.41; Adjusted R^2^ = 0.39.

**Table 8:** Correlation between Quality of Life and Multiple Sclerosis clinical parameters / patient-reported outcomes.

Parameter	r^S^	*P*
EQ-5D	0.824	< 0.001
MFIS physical	0.802	< 0.001
EDSS	0.625	< 0.001
MFIS total	0.374	< 0.001
Age	0.021	0.640
Year of diagnosis	0.004	0.823

EuroQol Questionnaire; MFIS, Modified Fatigue Impact Scale; EDSS, Expanded Disability Status Scale; r^S^: (0–0.11 weak correlation, 0.12–0.22 moderate correlation, 0.23–1 strong correlation).

### 3.3 Impact of health crisis across different healthcare provision settings

The COVID-19 pandemic introduced significant challenges to healthcare delivery, particularly for patients with chronic conditions like MS. This study explored how specialized MS centers adapted to these challenges and the impact of the pandemic on patient care and outcomes.

#### 3.3.1 Adaptation of healthcare services

Participants followed in specialized MS centers reported fewer difficulties accessing healthcare services, suggesting greater adaptability of these structures during the pandemic during the pandemic, swiftly implementing remote care solutions to ensure continuity of care. According to the COVID-19 Impact Questionnaire, 75% of patients in specialized centers reported no difficulty to access a doctor/other healthcare professional related to Multiple Sclerosis due to the pandemic, compared to only 5% of patients in private practices (*p* < 0.001) (Table 9). Additionally, 76% of patients in specialized centers stated that they received adequate care despite the crisis, whereas only 49% of patients in private practices reported feeling adequately supported (*p* < 0.05) (data not shown). This indicates that specialized MS centers were more proactive in adopting alternative care models to meet patient needs during the crisis.

**Table 9:** Barriers to access the health system (visit to a doctor or health professional for issues related to Multiple Sclerosis) during the pandemic across different healthcare provision settings and during the pandemic by period (pre- and post- vaccination era).

Q: Due to the novel coronavirus, have you recently faced obstacles in visiting a doctor or healthcare professional for issues related to Multiple Sclerosis?				
Answer	MS center, *N* (%)	Private doctor/clinic, *N* (%)	Total	*p*-value
Yes	88 (25%)	396 (93%)	484 (62%)	< 0.001
No	260 (75%)	22 (5%)	282 (36%)	
N/A	0 (0%)	10 (2%)	10 (2%)	
Total	348 (45%)	428 (55%)	776	
	Pre- vaccination era *N* (%)	Post- vaccination era *N* (%)	Total *N*	*p*-value
Yes	108 (26%)	46 (13%)	154 (20%)	< 0.001
No	277 (67%)	307 (85%)	584 (75%)	
N/A	28 (7%)	10 (3%)	38 (5%)	
Total	413 (53%)	363 (47%)	776	

MS, Multiple Sclerosis; N, number; N/A, non-available. Percentages denote proportion over total N per healthcare provision setting and per period (pre - and post - vaccination era).

The distribution of responses was also studied with regard to the pandemic period upon which they were provided, and more specifically upon pre- and post-vaccination against SARS-CoV-2 era. A statistically significant difference (*p* < 0.001) was observed in access to a doctor or health professional for MS-related issues depending on the period, with the period before vaccination being associated with a significantly higher frequency of difficulty in access, compared to the post-vaccination era 26 (108/413) vs. 13% (46/363), respectively (Table 9). These differences may partly reflect temporal changes in healthcare system functioning during the course of the pandemic, rather than differences attributable solely to healthcare provision settings. Moreover, a significant proportion of patients (47%, *N* = 363) experienced difficulty accessing disease-modifying treatment for MS due to the pandemic (data not shown). It is noted that the majority of patients (58%, *N* = 247/428) who experienced difficulty accessing disease-modifying treatment for MS were patients who were monitored outside the MS Center. In contrast, 93% (*N* = 324/348) of patients followed by an MS Center reported no difficulty in accessing medication for MS and its effects (Table 10a). Regarding access to medication during the pandemic period, no differentiation was observed regarding the timing of the pandemic, specifically before and after the COVID-19 vaccination phase (data not shown). Overall, regarding the ability of patients to perform the necessary tests required for MS-related issues, it appears that a large percentage overall (75%, *N* = 581/776) had access, despite the pandemic. Only 20% (*N* = 156/776) stated that they faced difficulty accessing tests. It is important to mention that of the patients who were followed by a private practice/ other Neurological Clinic, the majority reported that they faced some obstacle in carrying out their necessary tests due to the pandemic, (50%, *N* = 213/428). Conversely, 91% (*N* = 318/248) of the patients who were being followed up at an MS Center stated that they did not face any difficulty in accessing necessary tests (Table 10b). The analysis was also performed based on the period of the pandemic, before and after the start of vaccinations. A statistically significant difference (*p* < 0.001) was observed in access to tests for the disease depending on the period of the pandemic, with the frequency of difficulty accessing tests being significantly higher in the pre-vaccination era (27%), compared to the post-vaccination era (12%) (data not shown).

**Table 10:** Barriers to access to (a) DMTs for MS and concomitant treatments and (b) laboratory tests (other than MRI) for MS due to the pandemic, across different healthcare provision settings.

Answer	MS center *N* (%)	Private doctor/clinic *N* (%)	Total *N*	*p*-value
(a) Q: Due to the novel coronavirus, have you recently faced obstacles in accessing medications for Multiple Sclerosis and its effects?				
Yes	23 (7%)	247 (58%)	270 (35%)	< 0.001
No	324 (93%)	172 (40%)	496 (64%)	
N/A	1 (0%)	9 (2%)	10 (1%)	
Total	348	428	776	
(b) Q: Due to the new coronavirus, have you recently faced obstacles in getting laboratory tests (other than MRI) for Multiple Sclerosis?				
Yes	27 (3%)	213 (50%)	240 (31%)	< 0.001
No	318 (91%)	179 (42%)	497 (64%)	
N/A	3 (1%)	36 (8%)	39 (5%)	
Total	348	428	776	

DMTs, disease-modifying treatments; MS, Multiple Sclerosis; MRI, magnetic resonance imaging; N/A, non-available. Percentages denote proportion over total N per healthcare provision setting.

#### 3.3.2 Correlation between care and outcomes across different healthcare provision settings

In order to quantify the relationship between the type of care received and patient outcomes, a multiple linear regression model was developed. The regression model included demographic and disease-related characteristics to account for potential confounding when evaluating the association between healthcare provision setting and patient-reported outcomes. The analysis revealed that the type of healthcare structure (specialized center vs. general setting), along with patient demographics (age, education level) and frequency of follow-ups, were significant predictors of patient outcomes, including quality of life and disease progression (*p* < 0.05). Spearman correlation analysis demonstrated strong associations between QoL and EQ-5D, MFIS Physical, and EDSS scores, whereas demographic variables such as age and year of diagnosis showed weak and non-significant correlations with QoL. Specifically, being followed in a specialized MS Center was associated with a 12.5% increase in quality of life (EQ-5D) scores compared to those followed in general healthcare settings. Additionally, patients with more frequent follow-ups (≥2 times per year) showed a 9.3% improvement in their quality of life scores compared to those with fewer follow-ups. Higher education levels were also a significant predictor, with university graduates reporting an 8.1% increase in their quality of life scores compared to those with lower education levels. Conversely, the presence of comorbidities was associated with a 14.7% reduction in quality of life (*p* < 0.05).

Patients followed in specialized MS centers and reporting more frequent follow-ups tended to report better outcomes across the measured indicators. Additionally, younger patients and those with higher levels of education tended to report a better quality of life, suggesting that demographic factors influence how patients experience their care. Specifically, patients monitored at specialized MS centers reported an average EQ-5D score of 0.78, compared to 0.64 for those followed in general healthcare settings (*p* < 0.05). Patients with higher education levels (university degree or higher) had an 8.1% increase in their quality of life scores compared to those with lower education levels (*p* < 0.05). Conversely, the presence of other chronic conditions was negatively correlated with quality of life, with patients reporting a 14.7% reduction in their EQ-5D scores when comorbidities were present (*p* < 0.05).

The complete regression results, including adjusted effect sizes (β coefficients) and corresponding 95% confidence intervals, are presented in Table 7.

Overall, these findings underscore the importance of specialized care structures in maintaining patient wellbeing during healthcare crises. Specialized MS centers' ability to adapt quickly to changing circumstances and continue providing essential services helped mitigate the negative impact of the pandemic on MS patients.

## 4 Discussion

The present study sheds light on the role that specialized Multiple Sclerosis (MS) centers may play in the management of chronic diseases such as MS, particularly during healthcare crises like the COVID-19 pandemic. The findings indicate that patients who receive care at specialized MS centers reported better access to healthcare services and higher quality-of-life scores compared with those treated in general healthcare settings. Qualitative findings provided contextual interpretation of the quantitative results, offering additional insight into patient experiences regarding access to care and continuity of healthcare services during the pandemic. Furthermore, the study highlights the adaptability of specialized MS centers in times of crisis. By swiftly implementing remote care solutions, these centers ensured continuity of care during the pandemic, a crucial factor in maintaining patient health and wellbeing. The results of this study underscore the importance of specialized care structures in managing chronic diseases and suggest that healthcare systems should prioritize the development and support of such Centers.

One of the most significant findings of this study is the effectiveness of specialized MS centers in providing comprehensive care for MS patients. Those treated in these centers consistently reported better access to necessary healthcare services, including access to healthcare professionals, MRI scans, other laboratory investigations, DMTs and other treatments and consultations with other medical specialists. Although patients followed in specialized MS centers reported contact with a greater number of healthcare professionals, this measure reflects healthcare utilization rather than the formal structure or coordination of multidisciplinary teams. Therefore, the results should not be interpreted as direct evidence of coordinated multidisciplinary care but rather as an indication of broader access to different types of healthcare services within specialized care settings. This aligns with existing research, which indicates that specialized centers are better equipped to offer tailored care that meets the unique needs of patients with chronic conditions like MS. The multidisciplinary approach employed by MS Centers—integrating neurology, rehabilitation, and psychological support—proved essential for managing the complex and unpredictable nature of MS (5). Specialized care seems to enable earlier interventions, more accurate diagnoses, and more consistent monitoring of disease progression (19–21). In our study, although not specifically addressed, these factors likely contributed to the lower hospitalization rates and better disease management observed among patients treated at specialized MS centers. Moreover, the higher levels of patient satisfaction reported by those receiving care at specialized centers is a crucial indicator of the success of healthcare interventions. Research has shown that patient satisfaction is linked to better adherence to treatment plans and improved health outcomes, further reinforcing the value of specialized care (22).

The COVID-19 pandemic posed unprecedented challenges to healthcare systems worldwide, particularly in managing chronic diseases, such as MS (23). The study results highlight that patients faced barriers to accessing care during the pandemic, including delays in receiving routine services and reduced in-person visits, and heightened psychological distress due to uncertainty about their health. These findings are consistent with global reports highlighting the negative impact of the pandemic on chronic disease management (23). However, the study also revealed that specialized MS centers are better positioned to adapt to these challenges. Their rapid adoption of telemedicine and other remote care options allow patients to continue receiving essential services without compromising their safety. This adaptability underscores the resilience of specialized care structures in times of crisis and suggests that healthcare systems should invest in infrastructure that supports remote care options during future emergencies (24). The effectiveness of telemedicine in maintaining continuity of care during the pandemic aligns with previous research demonstrating that remote care options can effectively replace in-person visits for routine MS care (25). Patients express high satisfaction with remote care options services, citing the convenience and reduced need for travel as significant benefits (25, 26). These findings suggest that telemedicine and other remote care options should be integrated into routine MS care, even beyond the context of a health crisis. It should also be noted that improvements observed during the post-vaccination period may partly reflect the gradual normalization of healthcare system functioning as the pandemic evolved. Therefore, these differences should not be interpreted as evidence of structural superiority of specific healthcare settings but rather as part of broader temporal changes in healthcare delivery during the pandemic.

The findings of this study have important implications for healthcare policy, particularly in the management of chronic diseases, such as MS. The superior outcomes observed in specialized MS centers suggest that healthcare systems should prioritize the development and maintenance of such centers to provide optimal care for chronic disease patients (27, 28). Policymakers should consider allocating resources to expand access to specialized care, particularly in regions where such services are limited. Additionally, the study highlights the need for healthcare systems to be flexible and adaptable during crises (29). The success of telemedicine in maintaining continuity of care during the COVID-19 pandemic suggests that healthcare policies should support the integration of telemedicine into routine care for chronic disease patients (30). This could involve investments in telemedicine and other remote care infrastructure, training for healthcare professionals, and ensuring that patients have access to the technology needed for remote care. The study also underscores the importance of patient-centered care in healthcare policy (31). Patients' perceptions of the care they receive are critical for evaluating the effectiveness of healthcare interventions, and their feedback should be incorporated into healthcare planning and policy development (32). Ensuring that healthcare services meet the needs of patients, particularly during crises, can lead to better health outcomes and higher levels of patient satisfaction (5).

While this study provides valuable insights into the role of specialized MS centers and the impact of healthcare crises on chronic disease management, further research is needed to build on these findings. One important area for future research is the long-term impact of continuous care in specialized centers. Longitudinal studies that track patients over extended periods could provide a more comprehensive understanding of how specialized care influences disease progression, quality of life, and healthcare costs over time. Another area for future research is the cost-effectiveness of specialized MS centers. While these centers provide comprehensive care that improves patient outcomes, they also require significant investment in infrastructure, staffing, and technology (33). Comparative studies examining the cost-effectiveness of specialized centers vs. general healthcare settings could provide valuable insights for healthcare policymakers in deciding where to allocate resources most efficiently.

While the methodology employed in this study was designed to be robust, certain limitations must be acknowledged. Given the cross-sectional observational design of the study, the findings should be interpreted as associations rather than causal relationships. Furthermore, demographic differences between groups, particularly with regard to age and educational level, may have influenced some of the observed associations. Younger individuals and those with higher educational attainment may have greater access to healthcare resources and may report different perceptions of health-related quality of life (34). Although demographic characteristics were considered in the statistical analyses, the possibility of residual confounding cannot be completely excluded. Additionally, geographic accessibility to specialized MS centers may vary across regions, and individuals living in rural areas may face greater barriers in accessing specialized care. In addition, differences in baseline demographic characteristics and disease severity between healthcare provision settings may influence the observed associations. Younger patients and individuals with higher educational attainment may have different healthcare access patterns and may report different perceptions of health-related quality of life. Of note, education is a key social determinant of health, shown to affect MS outcomes and aspects of MS-related quality of life (35). Patients followed in specialized MS centers may also represent a heterogeneous population, including individuals referred for more complex disease management or treatment adjustments. These factors should be considered when interpreting the relationship between healthcare setting and patient-reported outcomes. The study was conducted in Northern Greece and it should also be noted that the findings of this study should be interpreted within the context of the Greek healthcare system. The organization of healthcare services, referral pathways, and reimbursement mechanisms may differ substantially across countries. Therefore, the observed patterns of healthcare access and utilization among people with Multiple Sclerosis may reflect characteristics specific to the Greek healthcare system. Consequently, caution should be exercised when generalizing these findings to other healthcare systems with different organizational structures or healthcare financing models. In addition, a large proportion of responses were collected through online questionnaires, and the study population included a relatively high proportion of participants with university-level education. This may introduce potential selection bias, as individuals with higher educational attainment or greater digital access may have been more likely to participate in the study. Furthermore, individuals with more severe disability or limited digital literacy may have been underrepresented in the online responses. Although efforts were made to provide both online and in-person participation options, the possibility of selection bias cannot be totally excluded. Therefore, the observed associations should be interpreted with caution when attributing differences solely to structural characteristics of healthcare provision. The reliance on self-reported data may introduce recall bias, particularly in the qualitative interviews. Although efforts were made to cross-reference patient records, the self-reported nature of the data remains a potential limitation. Additionally, the interpretation of hospitalization rates should be approached with caution. Patients referred to specialized MS centers may have undergone diagnostic procedures or hospitalizations in other healthcare settings prior to referral. As a result, hospitalization rates observed in this study may not fully reflect the quality of care within a specific healthcare setting but may instead reflect referral patterns and differences in patient pathways within the healthcare system.

In addition, several variables in the study relied on patient-reported information regarding healthcare access and perceived barriers to care. Although validated instruments were used to measure quality of life (EQ-5D) and disability status (PDDS), the questions related to healthcare access were study-specific items developed to capture patient experiences during the COVID-19 pandemic. As a result, these responses may be subject to recall bias or perception bias. Furthermore, the online completion of a large proportion of questionnaires may influence response patterns. These measurement limitations should therefore be considered when interpreting the results.

This research is significant for several reasons. First, MS is an increasingly prevalent condition that demands substantial healthcare resources. As the number of MS patients continues to rise (4), understanding the most effective care models becomes crucial for optimizing resource allocation and improving patient outcomes. Second, the COVID-19 pandemic has underscored the importance of resilient healthcare systems that can adapt to emergencies without compromising the care of vulnerable populations. By examining how specialized MS centers responded to the pandemic, this study provides important lessons for enhancing healthcare delivery during future crises. Additionally, the study emphasizes the importance of patient-centered care. MS has a profound impact on patients' lives, and their perceptions of the care they receive are critical for evaluating healthcare interventions (36, 37). This research highlights the value of incorporating patient feedback into healthcare policy and service development (32) to ensure that care models truly meet the needs of those they are designed to serve. By providing real-time data analysis of MS patient outcomes in Greece, the analysis fills a critical gap in the literature. It offers valuable insights for healthcare policymakers by examining how specialized centers adapted to the pandemic, ultimately informing strategies for managing chronic diseases during future crises.

To conclude, the study highlights a significant association between care in specialized MS centers and improved access to healthcare services and patient-reported outcomes, particularly during health crises such as the COVID-19 pandemic, by offering a comprehensive, multidisciplinary approach that addresses the multifaceted needs of MS patients. It highlights the necessity for adaptable healthcare systems to implement patient-centered strategies into chronic disease management to ensure continuity of care. Policymakers are encouraged to invest in remote care infrastructure and other remote care options for specialized centers, and to prioritize patient feedback in healthcare policy development. Further research is needed to explore the long-term impacts, cost-effectiveness, and best practices for remote care solutions in diverse contexts.

Although the findings highlight potential advantages of specialized care settings in the management of Multiple Sclerosis, the results should be interpreted cautiously when considering policy implications. The present study is observational and cross-sectional and does not include economic evaluations or cost-effectiveness analyses. Therefore, the policy implications discussed should be considered as hypotheses supported by associative evidence rather than definitive recommendations. Future research incorporating economic analyses and long-term outcome assessments would be required to inform healthcare policy decisions more robustly.

## Data Availability

The raw data supporting the conclusions of this article will be made available by the authors, without undue reservation.
